# Association between RBC transfusion and 1-year mortality in ICU survivors

**DOI:** 10.1186/s13054-022-04239-y

**Published:** 2022-12-01

**Authors:** M. M. Shangzhong Chen, M. M. Huojun Jiang, M. M. Caibao Hu

**Affiliations:** 1 Department of Intensiventensive Care, Zhejiang Hospital, 1229 Gudun Road, Hangzhou, 310013 Zhejiang China; 2 Department of Intensiventensive Care, Suzhou Integrated Chinese and Western Medicine Hospital, Tongjing South Road 81#, Suzhou, Jiangsu China

To the Editor,

We read with great interest the recent study by Dr. Blet et al. [1], in which they investigated the association between red blood cell (RBC) transfusion and 1-year mortality in ICU survivors. Aiming to establish an unbiased causal inference, an augmented inverse probability of weighting (AIPW) approach based on an inverse-probability weighted (IPW) estimator was used [2]. In the doubly robust estimation, RBC transfusion was significantly associated with 1-year mortality. This study is well performed. However, several points should be noted.

Superior to traditional likelihood-based or IPW estimators, AIPW involved both two models: propensity score model for exposure variable (here RBC transfusion) and regression model for the outcome (here 1-year mortality) [2, 3]. The result of AIPW will be unbiased when either the propensity score model or regression model is correctly specified.

For accuracy estimation, proper confounder selection is essential in both propensity score and regression models [4]. However, in the current study, only the blue covariates were used for propensity score estimation (including CRP, IL-6, PCT, GALECTIN-3, NTproBNP, cystatine-c, Pngal, hsTnI). Noteworthy, these variables were only associated with 1-year mortality but not with RBC transfusion, which means these variables were balanced between RBC and Non-RBC transfusion groups. Thus, including these variables in the IPW estimation may be inappropriate. Instead, variables associated with RBC transfusion should be included in IPW, such as disease severity, hemoglobin level necessitating transfusion, reasons for hypo-hemoglobinemia, etc. Thus, there is a risk that the propensity score model for the exposure variable may be biased.

In the Cox regression model for 1-year mortality, all the blue and red variables were included. Although 40 variables were included, some critical factors were still missing. According to current transfusion guidelines, RBC transfusions were commonly initiated at a low RBC threshold (7–8 g/dL) [5]. Therefore, as a strong transfusion indication and a significant risk for mortality, the lowest hemoglobin level should be balanced in the propensity score model or adjusted in the Cox-regression model (if not, there is a risk that the association between RBC transfusion and mortality may be mediated by the lowest hemoglobin level: lowest hemoglobin leads to RBC transfusion, and lowest hemoglobin leads to high mortality, which generated the relationship between RBC transfusion and mortality). However, in the current study, only hemoglobin at inclusion was included in the Cox-regression model. Thus, although the AIPW approach was adopted, there is still a relatively high risk of bias due to inadequate confounder selection.

In addition, for interventions (RBC transfusion) with strong clinical indications (low hemoglobin level, massive bleeding) that are closely related to poor prognosis, observational studies are difficult to reach an unbiased result, due to the inherent relationships that arise from selective use. This may be the reason why most current guidelines based on randomized controlled studies recommend transfusion in a critical situation, whereas most observational studies have reported a positive association between RBC transfusion and increased mortality.

Beyond these points, Dr. Blet et al. has to be congratulated for their great work in ICU survivors.

## Data Availability

Not applicable.
